# R-CenterNet+: Anchor-Free Detector for Ship Detection in SAR Images

**DOI:** 10.3390/s21175693

**Published:** 2021-08-24

**Authors:** Yuhang Jiang, Wanwu Li, Lin Liu

**Affiliations:** College of Geodesy and Geomatics, Shandong University of Science and Technology, Qingdao 266590, China; jiangyuhangcn@163.com (Y.J.); liwanwuqd@126.com (W.L.)

**Keywords:** SAR image, ship detection, deep learning model, anchor-free detector, attention

## Abstract

In recent years, the rapid development of Deep Learning (DL) has provided a new method for ship detection in Synthetic Aperture Radar (SAR) images. However, there are still four challenges in this task. (1) The ship targets in SAR images are very sparse. A large number of unnecessary anchor boxes may be generated on the feature map when using traditional anchor-based detection models, which could greatly increase the amount of computation and make it difficult to achieve real-time rapid detection. (2) The size of the ship targets in SAR images is relatively small. Most of the detection methods have poor performance on small ships in large scenes. (3) The terrestrial background in SAR images is very complicated. Ship targets are susceptible to interference from complex backgrounds, and there are serious false detections and missed detections. (4) The ship targets in SAR images are characterized by a large aspect ratio, arbitrary direction and dense arrangement. Traditional horizontal box detection can cause non-target areas to interfere with the extraction of ship features, and it is difficult to accurately express the length, width and axial information of ship targets. To solve these problems, we propose an effective lightweight anchor-free detector called R-Centernet+ in the paper. Its features are as follows: the Convolutional Block Attention Module (CBAM) is introduced to the backbone network to improve the focusing ability on small ships; the Foreground Enhance Module (FEM) is used to introduce foreground information to reduce the interference of the complex background; the detection head that can output the ship angle map is designed to realize the rotation detection of ship targets. To verify the validity of the proposed model in this paper, experiments are performed on two public SAR image datasets, i.e., SAR Ship Detection Dataset (SSDD) and AIR-SARShip. The results show that the proposed R-Centernet+ detector can detect both inshore and offshore ships with higher accuracy than traditional models with an average precision of 95.11% on SSDD and 84.89% on AIR-SARShip, and the detection speed is quite fast with 33 frames per second.

## 1. Introduction

Synthetic aperture radar (SAR) is an active microwave sensor with high resolution and wide swath [1]. Because of the ability to provide all-day and all-weather images of the ocean environment, it has been widely used in marine surveillance and deformation monitoring [2,3]. As an important task of marine surveillance, ship detection in SAR images is attracting increasing attention.

SAR images are different from optical images due to the complex imaging mechanisms and speckle noises. Moreover, phase errors caused by topography variations lead to the degradation of the focusing quality and geometric distortion of High-Resolution SAR images [4]. The interpretation and understanding of SAR images are very difficult, so it is necessary to establish the automatic target recognition (ATR) system of SAR images [5]. Numerous SAR ship detection methods have been proposed in recent years. There are three traditional methods for ship detection, including threshold methods [6], statistics methods [7], and transformation methods [8]. Among these methods, the constant false alarm rate (CFAR) and its improved versions [9,10,11,12,13,14,15] have been extensively studied and applied. However, the CFAR performs poorly in small object detection and complex scene detection. It cannot meet the requirements of high-precision ship detection.

Due to the powerful ability of feature extraction and expression, Convolution Neural Network (CNN) has become the mainstream approach for object detection [16]. Currently, object detection methods based on of Deep Learning (DL) can be roughly divided into two categories. The first is anchor-based methods such as Faster Region-CNN (R-CNN) [17], Mask R-CNN [18], Single Shot MultiBox Detector (SSD) [19] and RetinaNet [20]. Anchor-based methods introduce a Region Proposal Network (RPN) to generate a large number of candidate regions where targets may be included. Then, the candidate regions are classified and turned by CNN to obtain bounding boxes. This kind of method has relatively high accuracy with huge time consumption. The second is anchor-free methods such as You Only Look Once v1 (YOLOv1) [21], CornerNet [22], CenterNet [23] and Fully Convolutional One-Stage (FCOS) [24]. Anchor-free methods do not generate candidate regions in advance, and directly regress the category and location of bounding boxes. This kind of method is relatively fast and easy to train. This means they are more suitable for real-time processing and mobile deployment.

The number of available SAR images has increased greatly in recent years, which makes it possible to use the DL method for SAR ship detection. More and more scholars use DL methods to detect ships in SAR images. Most of the studies are based on anchor-based detection methods. For example, Li et al. [25] proposed an improved faster R-CNN method and released an SAR Ship Detection Dataset (SSDD). Kang et al. [26] came up with a contextual region-based CNN with multilayer fusion to rule out false alarms. Jiao et al. [27] improved faster R-CNN with Feature Pyramid Networks (FPN) to detect multiscale ships. A dense attention pyramid network was invented to detect dense small ships [28]. Zhao et al. [29] proposed a cascade coupled convolutional network with an attention mechanism to alleviate the problem of missing detections for small and densely clustered ships. Wang et al. [30] applied an object detector based on RetinaNet in multi-resolution SAR images. Wang et al. [31] combined SSD with transfer learning to address ship detection in complex scenes. Mao et al. [32] put forward an efficient SAR ship detector based on simplified U-Net.

In order to realize real-time detection, more and more methods focus on the high-speed processing of SAR ship detection. What is significantly different from other images is that ship targets are very sparse in SAR images. Considering this characteristic of SAR images, some scholars use anchor-free methods for SAR ship detection to accelerate the SAR image processing speed. Gao et al. [33] designed an anchor-free detector with lightweight backbone MobileNetV2. Guo et al. [34] improved CenterNet with a feature refinement module, feature pyramids fusion module and head enhancement module.

Although the above studies show that the DL methods can be applied in SAR ship detection, there are still four challenges affecting its performance: Heavy time cost, small ship detection, complex background and lack of accurate ship size and axial information. In order to solve the above problems, we propose an effective anchor-free detector for SAR ship detection called R-CenterNet+. First, to improve the focusing ability on small ships, we introduce an attention mechanism to the backbone network. Second, to distinguish between ships and complex background, FEM is employed to refine the feature map. Finally, we adopt the rotating bounding box to annotate ships and add the angle regression branch to the original detection head to predict ships’ rotation angle.

The main contributions of our work in this paper are as follows:

(1) For small ship detection, the feature extraction network Convolutional Block Attention Module Deep Layer Aggregation 34 (CBAM-DLA34) is established, which improves the performance of small ship detection;
(2) For the complex background, we adopt FEM to improve the robustness of ship detection in a complex background;
(3) We realize the rotation detection of ship targets to extract accurate ship size and axial information;
(4) The annotations based on rotating bounding box of SSDD and AIR-SARShip are established to evaluate the proposed model; and
(5) We conduct extensive experiments on SSDD and AIR-SARShip. The results show that our R-Centernet+ detector is an effective detector for both inshore and offshore ships, which is superior to the traditional models in accuracy and speed.

## 2. Data

### 2.1. SSDD Dataset

#### 2.1.1. Dataset Introduction

The SSDD dataset, established in 2017, is the first published dataset specifically for ship target detection in SAR images at home and abroad [25]. The SSDD dataset contains various images of different sensors, resolutions, polarizations, and scenes, etc. Using the production process of the PASCAL VOC dataset for reference, the SSDD dataset can be used to train and test ship detection algorithms, which enables researchers to compare algorithm performance under the same conditions. The detailed descriptions of the SSDD dataset are shown in Table 1.

#### 2.1.2. Dataset Processing

(1) Ship target classification

**Figure 1:**
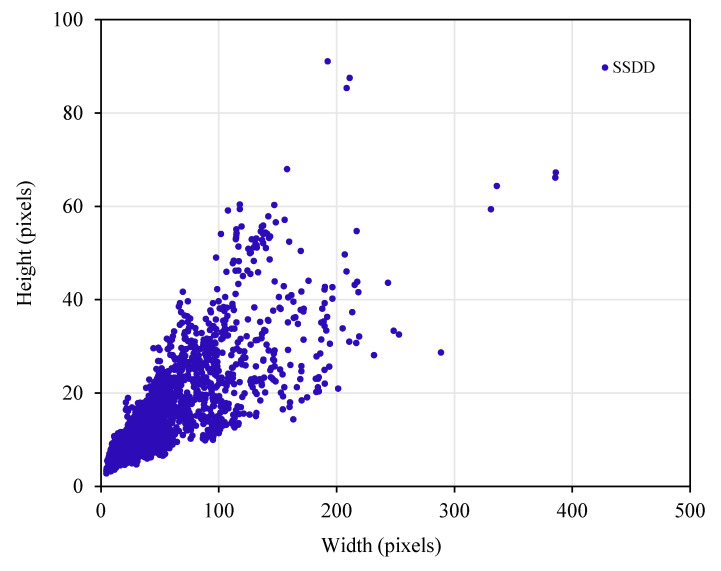
Distribution of ship size on SSDD.

The distribution of ship pixel size on the SSDD dataset is shown in Figure 1. In Figure 1, we measure ship size from the pixel level (i.e., the number of pixels) rather than the physical level. The reasons are as follows: (1) SAR images in different datasets have inconsistent resolutions, and these datasets’ publishers only provided a rough resolution range, so we cannot perform a strict comparison using the physical size; (2) in the deep learning community, it is common sense to use pixels to measure the object size in a relative pixel proportion among the whole image [35]. As can be seen from the Figure 1, the SSDD dataset is mainly composed of small ships. Moreover, the ship size varies greatly, with the smallest being about 7 × 4 pixels and the largest being about 381 × 69 pixels, which increases the diversity of ships on the dataset.

In order to improve the detection performance of the proposed model for multi-scale ships, we classify the ship targets on the SSDD dataset by ship pixel size. We classify ship objects with an area less than 32 × 32 pixels as small ships, with an area from 32 × 32 pixels to 64 × 64 pixels as medium-sized ships, and with an area larger than 64 × 64 pixels as large ships. The statistical results of ship targets are shown in Table 2.

(2) Inshore and offshore dataset classification

In general, it is difficult to detect inshore ships due to the interference of complex backgrounds [28]. In order to effectively reduce the interference of complex backgrounds and better detect inshore ships, we regard images containing land as inshore samples, and images not containing land as offshore samples, and then establish SSDD-inshore and SSDD-offshore datasets, which are shown in Table 3.

(3) Oversample of local image data

**Figure 2:**
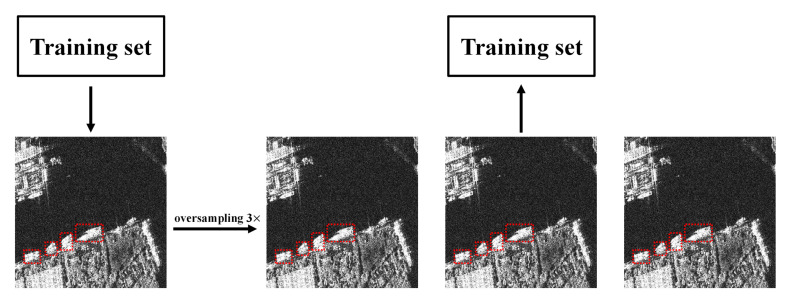
Variation of loss during training on the SSDD dataset.

The SSDD dataset contains some densely arranged small ships, which are relatively few compared to larger-sized ships. This imbalance of data distribution will cause the model to pay more attention to larger-sized ships, which will confuse multiple densely arranged small ship targets with one larger ship target, resulting in serious missed detections. In order to improve the detection performance, during the training process, we perform oversample data enhancement on images containing densely arranged small ships to encourage the model to pay more attention to them. The method of the oversample is shown in Figure 2.

### 2.2. AIR-SARShip Dataset

#### 2.2.1. Dataset Introduction

The AIR-SARShip dataset, established in 2019, is a SAR ship detection dataset of high resolution and large-size scene [36]. The dataset contains 31 large-scale SAR images of Gaofen-3 (GF-3). The scene types include ports, islands and reefs, sea surface with different levels of sea state, etc. The background covers various scenes such as inshore and offshore scenes. The AIR-SARShip dataset uses the production process of PASCAL VOC dataset for reference. The detailed descriptions of AIR-SARShip are shown in Table 4.

#### 2.2.2. Dataset Processing

(1) Image cropping into sub-images

Taking into account the limitation of Graphics Processing Unit (GPU) memory, we crop 31 large images into sub-images of 512 × 512 pixels in steps of 256 pixels. In order to reduce the imbalance between the foreground and background of the dataset, only reserve sub-images containing ship targets were used for training and testing. In the end, the cropped AIR-SARShip dataset includes a total of 719 sub-images and 1585 ship targets. This kind of image cropping is common sense in the DL community, and the original large SAR images with 24,000 × 16,000 pixels in the LS-SSDD-v1.0 dataset [37] are cropped into small sub-images with 800 × 800 pixels for the neural network training.

(2) Ship target classification

**Figure 3:**
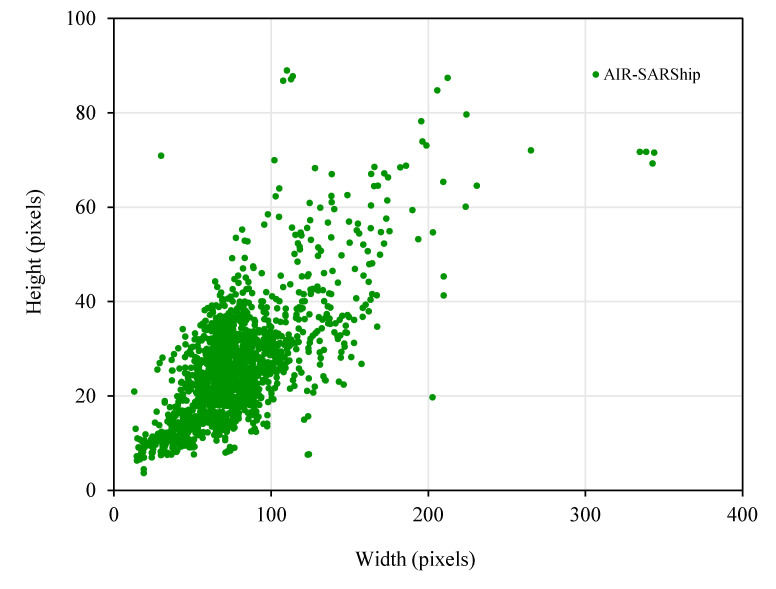
Distribution of ship size on AIR-SARShip.

The distribution of ship pixel size on the cropped AIR-SARShip dataset is shown in Figure 3. As can be seen from the figure, the AIR-SARShip dataset is mainly composed of small- and medium-sized ships. Ship size varies greatly, with the smallest being about 18 × 4 pixels and the largest being about 344 × 72 pixels.

Similar to the processing of the SSDD dataset, we classify ship targets of the cropped AIR-SARShip dataset by size. The results are shown in Table 5.

(3) Inshore and offshore dataset classification

We establish AIR-SARShip-inshore and AIR-SARShip-offshore datasets, as shown in Table 6.

## 3. Materials and Methods

**Figure 4:**
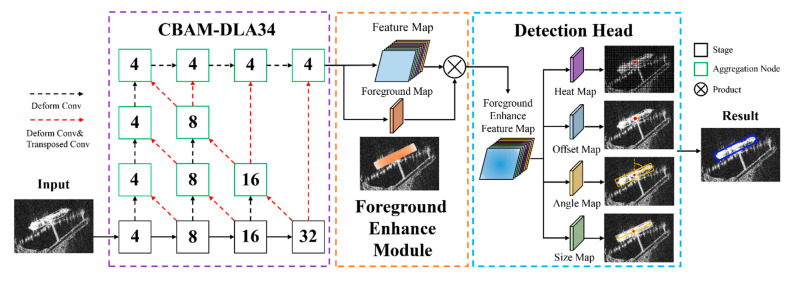
The architecture of R-Centernet+, which mainly consists of the CBAM-DLA34, FEM, and Improved Detection Head. The numbers in the boxes represent the stride to the image.

The proposed model is an anchor-free detector based on Centernet, and its overall framework is shown in Figure 4. The target detection process is as follows: first, use the backbone network of CBAM-DLA34 to extract ship features; then, input the original feature map into FEM and output the foreground enhancement feature map; finally, conduct regression for the center point coordinates, size, offset, and rotation angle of the ship, so as to realize the rotation detection of ship targets.

### 3.1. CBAM-DLA34

The imaging mechanism of SAR images is quite different to that of ordinary optical images. There are a lot of speckle noises, which makes it difficult to extract and merge features of SAR images, and leads to a weak focus on small ship targets. Therefore, we introduce the lightweight attention module CBAM [38] into the backbone network to build CBAM-DLA34. CBAM is an attention module that combines channel and space. It infers attention weights along two independent dimensions of the channel and space in turn, and then multiplies the attention weights with the input feature map for adaptive feature optimization. The attention module improves the feature expression ability of the feature extraction network for ship targets in SAR images, and improves the focus on small-scale ship targets.

The specific implementation of the channel attention module includes three steps.

(1) Perform global average pooling and maximum pooling of the spatial dimension on the input feature F of size  H×W×C, respectively, and obtain two feature maps of  1×1×C;

(2) Through multilayer perceptron (MLP), add the features output by the perceptron element by element; and

(3) Obtain the channel attention weight $M_{c}$ through Sigmoid activation function. It is shown in formula (1):

(1) $$M_{c}\left(F\right)=\sigma \left(MLP\left(AvgPool\left(F\right)\right)+MLP\left(MaxPool\left(F\right)\right)\right)\;\;=\sigma \left(W_{1}\left(W_{0}\left(F_{avg}^{c}\right)\right)+W_{0}\left(W_{1}\left(F_{max}^{c}\right)\right)\right)\;$$

In the formula, σ represents Sigmoid activation function,$\;W_{0}$ and $W_{1}$ represent the weight of MLP, $\;F_{avg}^{c}$ and $F_{max}^{c}$ represent the features output by global average pooling and maximum pooling, respectively.

The specific implementation of the spatial attention module is as follows.

(1) Perform global average pooling and maximum pooling of the channel dimension on the input feature $F^{\prime }$, respectively, and obtain two feature maps of size  H×W×1.

(2) Splice the feature maps together according to the channels, and perform the convolution operation through a 7×7 convolution layer; and

(3) Obtain the spatial attention weight $M_{s}$ through Sigmoid activation function. It is shown in formula (2):

$$M_{s}\left(F\right)=\sigma \left(f^{7\times 7}\left(\left[AvgPool\left(F\right);MaxPool\left(F\right)\right]\right)\right)$$

(2) $$=\sigma \left(f^{7\times 7}\left(\left[F_{avg}^{s};F_{max}^{s}\right]\right)\right)$$

In the formula, σ represents Sigmoid activation function, $f^{7\times 7}$ represents a convolution operation with the filter size of  7×7, $F_{avg}^{s}\;$ and $F_{max}^{s}\;$ represent the features output by global average pooling and maximum pooling, respectively.

**Figure 5:**
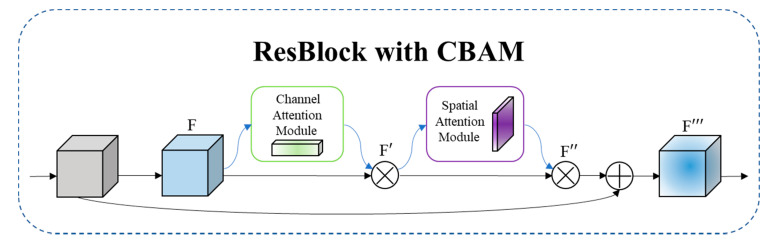
The overview of CBAM. The module has two sequential sub-modules: channel and spatial. The intermediate feature map is adaptively refined through CBAM at every ResBlock of DLA34.

The method of adding CBAM to ResBlock [39] is shown in Figure 5. First, perform a convolution calculation on the feature map generated by the previous residual block to generate an input feature map  F. Second, Input F into the channel attention module to obtain the channel attention feature $\;M_{c}$, and then obtain the feature map $F^{\prime }$ by multiplying *F* and $M_{c}$ element by element. Third, input $F^{\prime }$ into the spatial attention module to obtain the spatial attention feature $\;M_{s}$, and then obtain the feature map $F^{\prime \prime }$ by multiplying $F^{\prime }$ and $M_{s}$ element by element. Finally, perform a shortcut connection to obtain the final feature map $F^{\prime \prime \prime }$, which is used as the input of the next ResBlock.

DLA is a network with hierarchical skip connections [40]. It designs two structures: Iterative Deep Aggregation (IDA) and Hierarchical Deep Aggregation (HDA). According to the basic network structure, IDA refines the resolution and aggregation scale step by step to integrate semantic information. HDA aggregates various levels into representations of different grades through its own tree-like connection structure to integrate spatial information.

**Figure 6:**
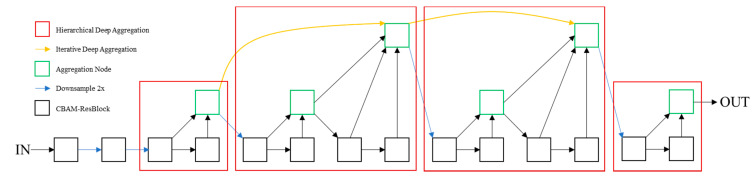
Structure of CBAM-DLA34.

We improve the DLA network, and propose CBAM-DLA34, which uses CBAM-ResBlock as the basic module. The specific structure of CBAM-DLA34 is shown in Figure 6.

### 3.2. FEM

Due to the special imaging environment, the background of the SAR image is very complicated. Ship targets are easily affected by islands, reefs and speckle noise, especially in the detection of inshore ships. The traditional DL model is easy to confuse the ship target with the background of the similar shape, resulting in false detections and missed detections. Studies [41,42,43] show that semantic segmentation can assist object detection. The R-Centernet+ detector model proposed in this paper adopts an FEM based on semantic segmentation to predict the foreground area in advance to reduce the interference of complex backgrounds.

**Figure 7:**
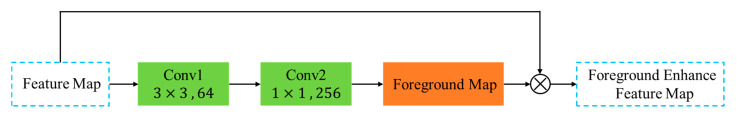
Pipeline of FEM.

The implementation of the FEM is shown in Figure 7. We perform continuous convolution on the original feature map extracted by the backbone network to obtain the foreground image *F*. Element-wise multiplication on the foreground image *F* and the original feature map *FM* generates a foreground enhanced feature map *FEFM*.

(3) FEFM=FM⊙F 

**Figure 8:**
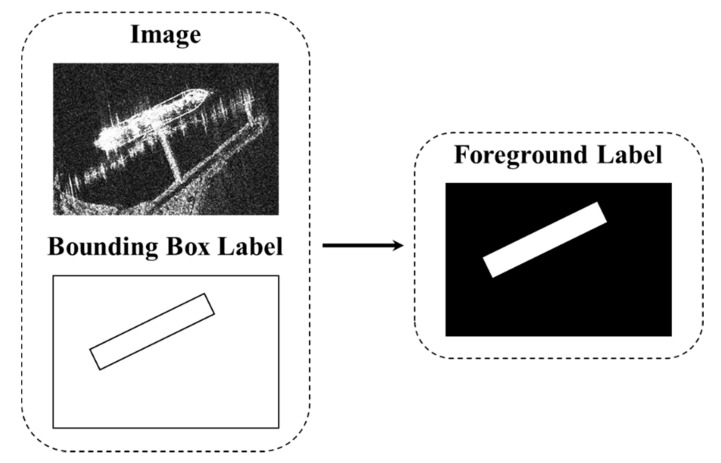
The schematic diagram of Foreground segmentation label generation: The Foreground segmentation labels are generated from the bounding box labels.

In order to mark the foreground accurately, the foreground labels based on semantic segmentation are used to train FEM. The generation method of foreground labels is shown in Figure 8. First, project the groundtruth bounding box of an SAR image with a width (W) and a height (H) to the corresponding position under the output stride of R. Then create a label of size $\;\frac{W}{R}\times \frac{H}{R}\times 1$. If the pixel is within the projected bounding box, set the value to 1, otherwise set the value to 0.

### 3.3. Improved Detection Head

**Figure 9:**
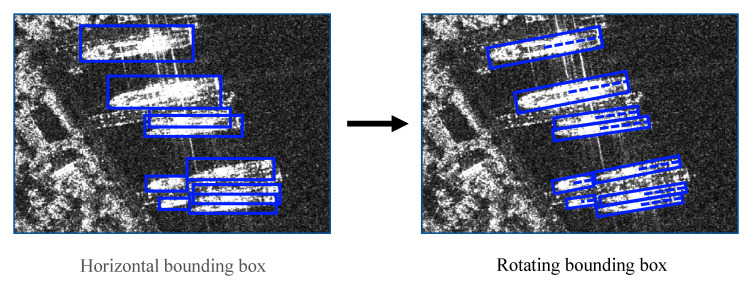
The difference between two kinds of labels.

Ship targets in SAR images are characterized by a large aspect ratio, arbitrary direction and dense arrangement. As shown in the left figure of Figure 9, the traditional horizontal box detection is difficult to accurately express the length, width and axial information of ship targets. More importantly, in complex scenes, the horizontal box contains a lot of non-target area information. Compared with the horizontal box, the rotating box can reflect the ship information more accurately, and avoid the interference of the non-target area on the ship feature extraction, which meets the actual requirements of ship target feature extraction in SAR images. In order to achieve the fine detection of ship targets, the detection head structure of the Centernet network is improved, and the output of the angle map is added on the original basis, which realizes the rotation detection of ship targets in SAR images.

An SAR image with a width of W and a height of H can generate four regression maps:

(1) In the branch of position regression, the center point heat map $P\in {\left[0,\;1\right]}^{\frac{W}{R}\times \frac{H}{R}\times C}$ is generated, where R is the scaling ratio of output size, and C is the number of object types in the ship detection (C=1). When $P_{xyc}=1$, it means the center point is detected. Gaussian kernel $Y_{xyc}=\exp\left(-\frac{\left(x-{\tilde{p}}_{x}\right)+{\left(y-{\tilde{p}}_{y}\right)}^{2}}{2\sigma_{p}^{2}}\right)$ is used to map each center point to the center point heat map, where $\tilde{p}=|\frac{p}{R}|$ is the center point corresponding to low resolution, and $\sigma_{p}$ is the standard deviation related to the target size. In the inference, the first 100 peaks in the heat map whose values are not less than 8 connected neighbors are used as the center points for prediction;

(2) In the branch of size regression, the size prediction map $\overset{\wedge }{s}\in R^{\frac{W}{R}\times \frac{H}{R}\times 2}$ is generated. For a center point $\;p_{k}$, there is a size prediction $\;S_{k}=(w_{k},h_{k})$, where $w_{k}$ and $h_{k}$ correspond to the width and height of the ship target, respectively;

(3) In the branch of center point offset regression, the center point offset prediction map $\overset{\wedge }{o}\in R^{\frac{W}{R}\times \frac{H}{R}\times 2}$ is generated. All the ship targets share the same offset prediction value $O_{k}=(\delta_{k},\delta_{k})$ to recover the discretization error caused by the output step size; and

(4) In the branch of angle regression, the angle prediction map $\overset{\wedge }{s}\in R^{\frac{W}{R}\times \frac{H}{R}\times 1}$ is generated. The angle prediction value $A_{k}=\theta_{k}$ corresponds to the rotation angle of a ship target.

**Figure 10:**
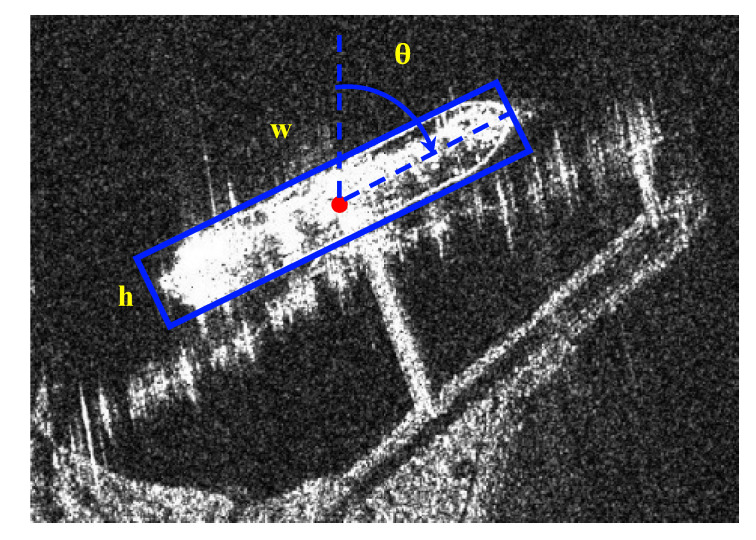
The schematic diagram of the rotating bounding box.

In order to realize the regression of the ship rotation angle, we use the rotating rectangular box to mark the ship target. In this paper, the method of the rotating rectangular box is defined as (x,y,w,h,θ), where (x,y) is the center point coordinate of the rotating rectangle, w is the long side of the rectangle, and h is the short side of the rectangle. As shown in Figure 10, θ represents the angle that the positive direction of y-axis rotates clockwise to the long side of the rectangle, and its range is [0, 180).

### 3.4. Loss Function

The loss function can be divided into five parts: ①$\;L_{f}$ is the loss of foreground prediction; ②$\;L_{p}$ is the loss of center point prediction; ③$\;L_{s}$ is the loss of size prediction; ④$\;L_{o}$ is the loss of center point offset prediction; ⑤$\;L_{a}$ is the loss of angle prediction.

$L_{f}$ and $L_{p}$ use the modified focal loss, as respectively shown in Formulas (4) and (5).

(4) $$L_{f}=\frac{-1}{N}\underset{xyc}{\sum }\{\begin{array}{c} {\left(1-F_{xyc}\right)}^{\alpha }log\left(F_{xyc}\right)\;\;\;\;\;G_{f_{xyc}}=1\\ {\left(1-G_{f_{xyc}}\right)}^{\beta }{\left(F_{xyc}\right)}^{\alpha }\;\;\;\;\;\;\;\;\;\;\;\;\;\;\\ log\left(1-F_{xyc}\right)\;\;\;\;\;\;\;\;\;otherwise \end{array}$$

In the formula, $F_{xyc}$ is the predicted value of the foreground point, $G_{f_{xyc}}$ is its groundtruth, N is the number of foreground points in the picture, and α and β are hyperparameters. According to [22] and our experiments before, we set α=2, β=4 in the experiments that result in the best outcomes.

(5) $$L_{p}=\frac{-1}{N}\underset{xyc}{\sum }\{\begin{array}{c} {\left(1-P_{xyc}\right)}^{\alpha }log\left(P_{xyc}\right)\;\;\;\;\;G_{p_{xyc}}=1\\ {\left(1-G_{p_{xyc}}\right)}^{\beta }{\left(P_{xyc}\right)}^{\alpha }\;\;\;\;\;\;\;\;\;\;\;\;\;\;\\ log\left(1-P_{xyc}\right)\;\;\;\;\;\;\;\;\;otherwise \end{array}\;$$

In the formula, $P_{xyc}$ is the predicted value of the center point, $G_{p_{xyc}}$ is its groundtruth, *N* is the number of center points in the picture, α and β are hyperparameters. We also set α=2, β=4.

$L_{s}$, $L_{o}$ and $L_{a}$ use the modified smooth L1 loss, as shown in formulas (6)–(8), respectively.

(6) $$L_{s}=\frac{1}{N}\overset{N}{\underset{k=1}{\sum }}\{\begin{array}{c} 0.5*{\left(S_{k}-G_{sk}\right)}^{2}\;\;\;\;if\left|S_{k}-G_{sk}\right|<1\\ \left|S_{k}-G_{sk}\right|-0.5\;\;\;\;otherwise \end{array}$$

In the formula, $S_{k}$ is the predicted value of the target size at the center point $P_{k}$, and $G_{sk}$ is its groundtruth.

(7) $$L_{s}=\frac{1}{N}\overset{N}{\underset{k=1}{\sum }}\{\begin{array}{c} 0.5*{\left(O_{k}-G_{ok}\right)}^{2}\;\;\;\;if\left|O_{k}-G_{ok}\right|<1\\ \left|O_{k}-G_{ok}\right|-0.5\;\;\;\;otherwise \end{array}$$

In the formula, $O_{k}$ is the predicted value of the center point offset at the center point $P_{k}$, and $G_{ok}$ is its groundtruth.

(8) $$L_{s}=\frac{1}{N}\overset{N}{\underset{k=1}{\sum }}\{\begin{array}{c} 0.5*{\left(A_{k}-G_{ak}\right)}^{2}\;\;\;\;if\;\left|A_{k}-G_{ak}\right|<1\\ \left|A_{k}-G_{ak}\right|-0.5\;\;\;\;\;otherwise \end{array}$$

In the formula, $A_{k}$ is the predicted value of the target rotation angle at the center point $P_{k}$, and $G_{ak}$ is its groundtruth.

The overall loss function is:

(9) $$L=\lambda_{f}L_{f}+\lambda_{p}L_{p}+\lambda_{s}L_{s}+\lambda_{o}L_{o}+\lambda_{a}L_{a}\;$$

In the formula,$\;\lambda_{f}$,$\;\lambda_{p}$, $\;\lambda_{s}$,$\;\lambda_{o}$, $\lambda_{a}$ are the weight coefficients of five parts of loss value, respectively.

## 4. Experiment and Analysis

We conduct experiments on two SAR image ship datasets SSDD and AIR-SARShip. The experiments are implemented in the Pytorch DL environment built on Windows10 system, with NVIDIA 2070 Super, CUDA v10.0, cuDNN v7.4.2.

### 4.1. Evaluation Metric

We use Precision (*P*), Recall (*R*), Average Precision (*AP*), Frame Per Second (*FPS*) and FLoating-point Operations (*FLOPs*) to evaluate the effect of ship detection. The calculation formulas of *P* and *R* are as follows [44]:

(10) $$P=\frac{TP}{TP+FP}\;$$

(11) $$R=\frac{TP}{TP+FN}$$

In the formula, *TP* means true positive, *FP* means false positive, and *FN* means false negative. Generally speaking, *P* and *R* are a pair of values that affect each other, so it is difficult to evaluate the overall performance. Therefore, this paper uses *AP* to evaluate the detection model more objectively, which is expressed as:

(12) $$AP=\int_{0}^{1}P\left(R\right)dR\;$$

Detection speed is an important indicator of whether the model can be applied to actual detection tasks, and it should be paid attention to as much as detection precision. In order to comprehensively evaluate the performance of the proposed model, *FPS* is introduced to evaluate the speed of model detection.

(13) $$FPS=\frac{Frames}{1s}\;$$

In the formula, *Frames* represents the number of pictures processed by the detection model per second.

The time complexity is an important evaluation metric of the deep learning model. It determines the training and prediction time of the model. If the complexity is too high, it will lead to a lot of time for model training and prediction, which cannot quickly verify the idea and improve the model, nor can it achieve rapid prediction. We use *FLOPs* to measure the time complexity of the deep learning model. *FLOPs* of convolutional layers and fully connected layers are expressed as:

(14) $$FLOPs_{conv}=\left(2\times C_{i}\times K^{2}-1\right)\times H\times W\times C_{o}\;$$

(15) $$FLOPs_{fc}=\left(2\times I-1\right)\times O$$

In the formula, $C_{i}$ represents input channel, K represents kernel size, H and W represent output feature map size, $C_{o}$ represents output channel, I represents input neuron numbers and O represents output neuron numbers.

### 4.2. Experiments on SSDD

For SSDD, 80% of the entire dataset is randomly selected as the training set, and 20% of the entire dataset as the test set. During the training process of the SSDD dataset, the input SAR image is transformed to 512 × 512 pixels. The Adam optimizer with 0.9 momentum and 1 × 10^−4^ weight decay is used for training. The number in the batch size is 8. The initial learning rate is set to 1.25 × 10^−4^, and the training epoch is 120. After 60 epochs of training, the learning rate is further attenuated to 1.25 × 10^−5^, and after 90 epochs, it is further attenuated to 1.25 × 10^−6^. We set $\lambda_{p}=1.0$, $\lambda_{s}=0.1$, and $\;\lambda_{o}=1.0$ according to the reference of [23] (Zhou X etc., 2019). We performed four experiments on $\lambda_{a}$ and $\lambda_{f}$ to determine the optimal weight assignments. The experimental results on different weight assignments are shown in Table 7. Finally, we set $\lambda_{p}=1.0$, $\lambda_{s}=0.1$, $\;\lambda_{o}=1.0$, $\lambda_{a}=0.1$, and $\lambda_{f}=1$ in all our experiments.

**Figure 11:**
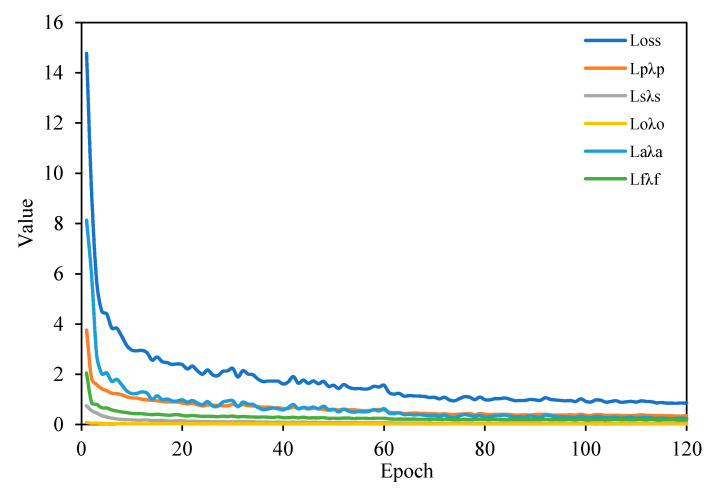
Variation of loss during training on the SSDD dataset.

In the training process, in addition to using an oversample, data enhancement methods such as random horizontal flipping and random cropping are also used. The loss changes during the training on the SSDD dataset are shown in Figure 11. As the number of training increases, the loss decreases constantly and the model converges.

**Figure 12:**
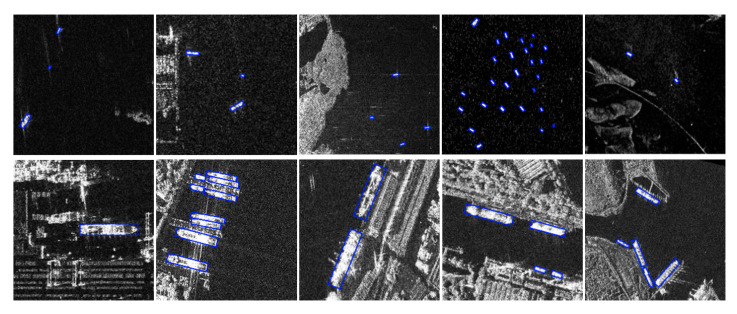
Satisfactory testing results of the SSDD dataset. Both small ships and ships in complex backgrounds can be detected correctly.

The satisfactory detection results on the SSDD dataset are shown in Figure 12. The first row of pictures shows the results of small ship detection, which is a challenging problem for ship detection. Obviously, the R-Centernet+ detector can effectively detect small ships. This shows that CBAM can improve the feature expression ability of the feature extraction network for ship targets, and enhance the focus on small ships. The second row of pictures shows the results of detecting ships in complex backgrounds. Obviously, R-Centernet+ detector can effectively distinguish ships from complex backgrounds. This shows that FEM is beneficial to distinguish the foreground and the background, and improve the performance of the detection model in complex scenes.

**Figure 13:**
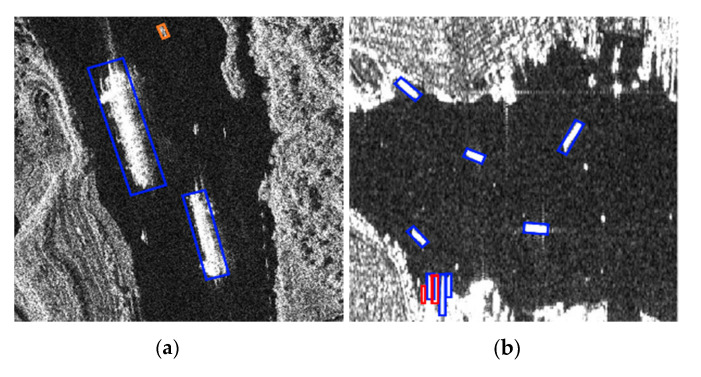
(**a**,**b**) Undesired results in the SSDD. Blue bounding box means ships that were detected, red bounding box means ships that were missed, and orange bounding box means false alarms.

However, there are still some false detections and missed detections in the SSDD dataset. As shown in Figure 13a, small ships close to each other are prone to missed detections. As shown in Figure 13b, a small number of small offshore islands are easily confused with ship targets.

**Figure 14:**
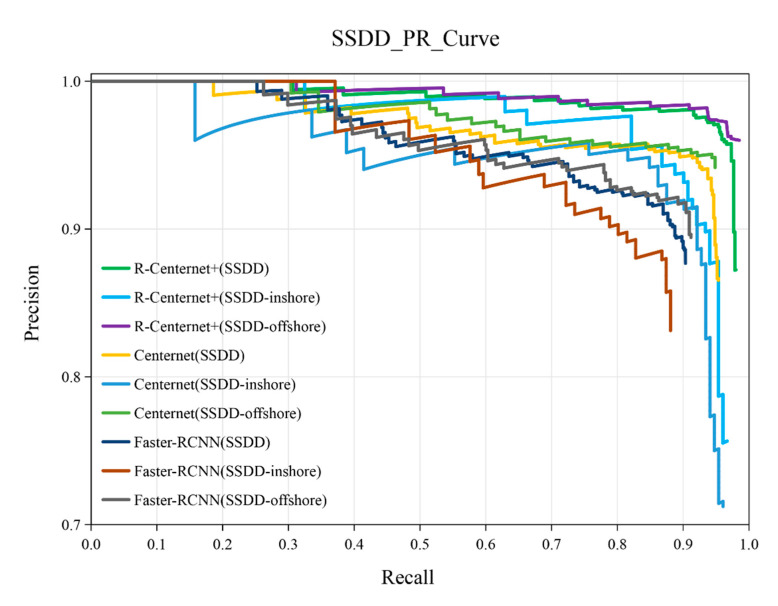
The P-R curves of the SSDD dataset.

The P-R curves of R-Centernet+, Centernet and Faster-RCNN on SSDD, SSDD-inshore and SSDD-offshore datasets are shown in Figure 14. It can be seen that both in inshore and offshore scenes, the P-R curves of the proposed method are relatively smooth, and the detection performance is better than the other two methods. The detailed experimental results on the SSDD dataset are shown in Table 8. The AP of the proposed method on the SSDD dataset reaches 95.11%, and the detection performance is quite great on the whole. The proposed R-Centernet+ detector can detect offshore ships of various scales well, and the AP of the proposed method on SSDD-offshore dataset reaches 97.84%. More importantly, this model can better detect inshore ships in complex backgrounds, and the AP value reaches 93.72%, which is superior to the Faster-RCNN and Centernet.

### 4.3. Experiments on AIR-SARShip

For the AIR-SARShip dataset, 21 of the 31 SAR images are used as the training set, and the remaining 10 are used as the test set. After image cropping, there are 474 sub-images in the training set and 245 sub-images in the test set. In the training process of the AIR-SARShip dataset, the input SAR image is transformed to 512 × 512 pixels. We use an Adam optimizer with 0.9 momentum and 1 × 10^−4^ weight decay for training. The number of the batch size is 8. The initial learning rate is set to 1 × 10^−4^, and the training epoch is 100. After 60 epochs of training, the learning rate further decays to 1 × 10^−5^, and after 80 epochs of training, it further decays to 1 × 10^−6^.

In the training process, the data enhancement methods of random horizontal flipping and random cropping are used.

**Figure 15:**
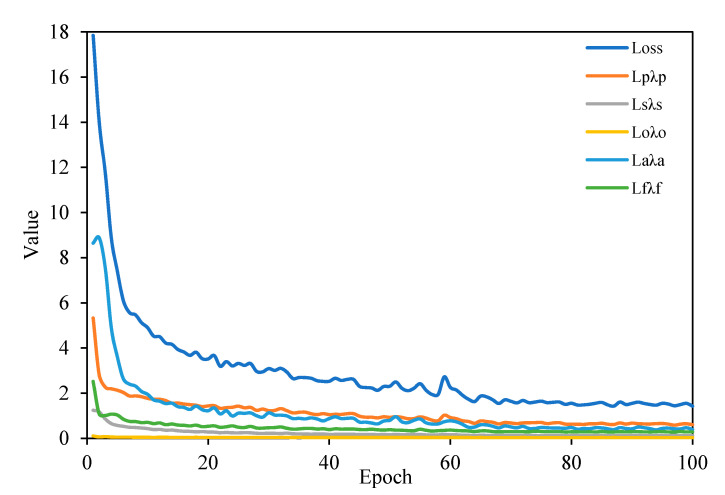
Variation of loss during training on the AIR-SARShip dataset.

The loss changes during the training on the AIR-SARShip dataset are shown in Figure 15. As the number of training increases, the loss decreases constantly and the model converges.

**Figure 16:**
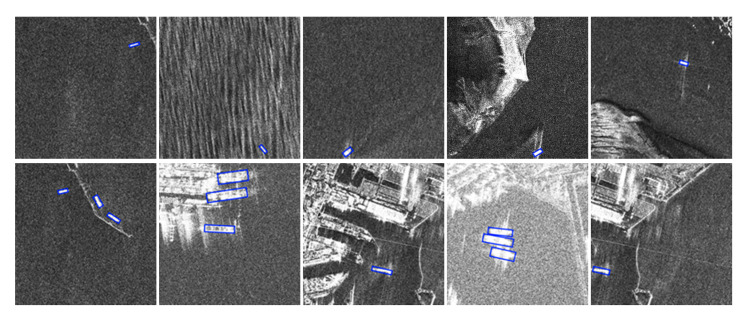
Satisfactory detection results on the AIR-SARShip dataset. Both small ships and ships in complex backgrounds can be detected correctly.

The satisfactory detection results on the Air-SARShip dataset are shown in Figure 16. The first row of pictures shows the results of detecting small ships, and the second row of pictures shows the results of detecting ships in complex backgrounds.

**Figure 17:**
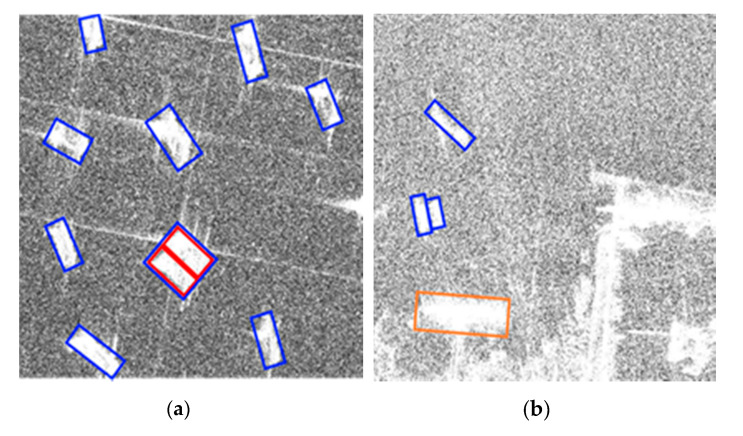
(**a**,**b**) Undesired result on the AIR-SARShip. Blue bounding box indicates the detected ships, red bounding box indicates the missed ships, and orange bounding box indicates false alarms.

Some false detections and missed detections on the AIR-SARShip dataset are shown in Figure 17a,b.

**Figure 18:**
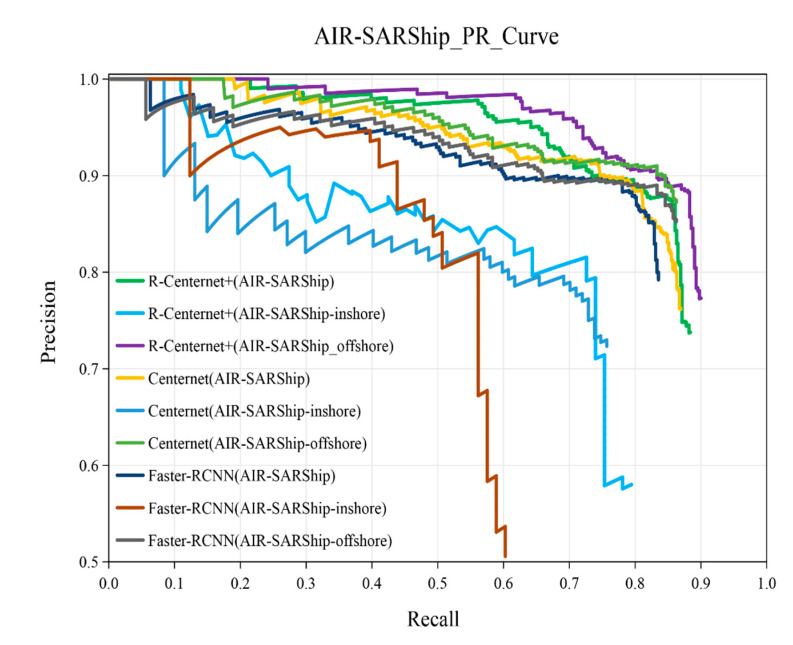
The P-R curves of AIR-SARShip dataset.

The P-R curves of R-Centernet+, Centernet and Faster-RCNN on AIR-SARShip, AIR-SARShip-inshore and AIR-SARShip-offshore datasets are shown in Figure 18. As shown in the figure, the detection performance of the proposed method is superior to the Faster-RCNN and Centernet. The detailed experimental results of the AIR-SARShip dataset are shown in Table 9. The AP of the proposed method on the AIR-SARShip dataset reaches 84.89%, which can be applied in actual detection task. The AP of the proposed method on the AIR-SARShip-offshore dataset reaches 87.43%, and on the AIR-SARShip-inshore dataset reaches 68.45%. On the AIR-SARShip dataset, the detection performance is lower than that on the SSDD dataset due to the great differences between the training set and the test set of the AIR-SARShip dataset. It is a challenging research topic to increase the migration ability of models in large detection scenes.

### 4.4. Comparisons with the State-of-the-Arts

We compare the R-Centernet+ detector with the state-of-the-art detection models on SSDD and AIR-SARShip datasets under the same conditions. The results of the comparisons are shown in Table 10. On the whole, the proposed R-Centernet+ detector is a stable and efficient SAR image ship detection model with high accuracy and fast speed.

First, we compare the proposed R-Centernet+ detector with the classic anchor-based detection model Faster-RCNN [14]. On the SSDD dataset, the AP of the proposed model is 95.11%, which is 6.51% higher than that of Faster-RCNN. On the AIR-SARShip dataset, the AP of the proposed model is 84.89%, which is 5.71% higher than that of Faster-RCNN. The main reason is that the center point can well represent the features of the ships, which has a great advantage over using the anchor box. And because there is no anchor and no Non-Maximum Suppression (NMS), the speed of the proposed method has been greatly improved, from 14 to 33 FPS. It indicates that the proposed R-Centernet+ detector shows great application value in real-time SAR ship detection.

The proposed R-Centernet+ detector is compared with the classic anchor-free detection model Centernet [20]. On the SSDD dataset, the AP of the proposed model is 1.29% higher than that of Centernet. On the AIR-SARShip dataset, the AP of the proposed model is 1.18% higher than that of Centernet. The main reason is that R-Centernet+ detector model adds the CBAM and FEM, which improves the detection performance of small ships and ships in complex scenes. In addition, the R-Centernet+ detector realizes rotation detection, reduces the interference of non-target areas on ship features extraction, and also increases the robustness of the detection model.

We also compare the number of the parameters, FLOPs and the model size of these methods. The results of the comparisons are shown in Table 11. It can be found from Table 11 that the addition of CBAM and FEM have little effect on the model parameters, FLOPs and model size. The number of parameters of the proposed method is low, which indicates it is a lightweight network. The model size of the proposed method is only 77.92 MB, and such a lightweight model is convenient for the future FPGA or DSP porting.

## 5. Discussion

CBAM and FEM are added to R-Centernet+ detector. In order to verify the effectiveness of each module, ablation experiments are performed on the SSDD dataset. The results of the ablation experiment are shown in Table 12.

### 5.1. Effectiveness of CBAM

**Figure 19:**
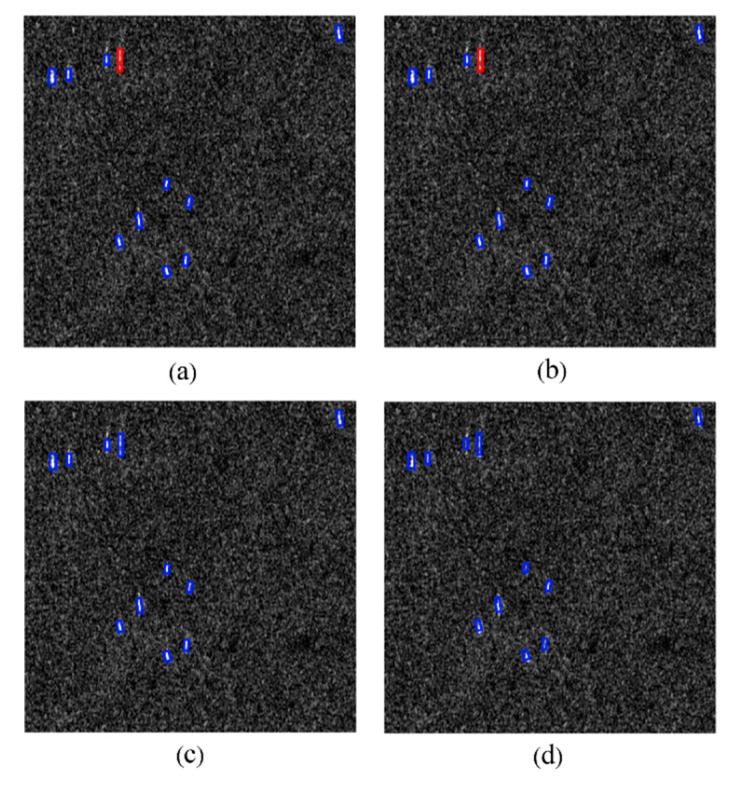
The effect of CBAM. (**a**) The detection result of the experiment setting of NO CBAM and NO FEM. (**b**) The detection result of the experiment setting of NO CBAM and YES FEM. (**c**) The detection result of the experiment setting of YES CBAM and NO FEM. (**d**) The detection result of the experiment setting of YES CBAM and YES FEM. The blue rectangles indicate detected ships. The red rectangles indicate missed ships.

In order to verify the effectiveness of the CBAM, a contrast experiment is performed on the SSDD dataset. The detection results of the selected typical scenes in which small ships assemble are shown in Figure 19. It can be seen from Figure 19 that the introduction of the attention mechanism can make it better to extract the features of small ships, thereby increasing the focus on small ships and reducing the missed detection rate of ships. Adding CBAM makes the performance of the R-Centernet+ detector better, and the AP increases by 0.80%. This illustrates the advantages of the CBAM method for ship detection in SAR images, especially for the small ships that are not easy to detect.

### 5.2. Effectiveness of FEM

**Figure 20:**
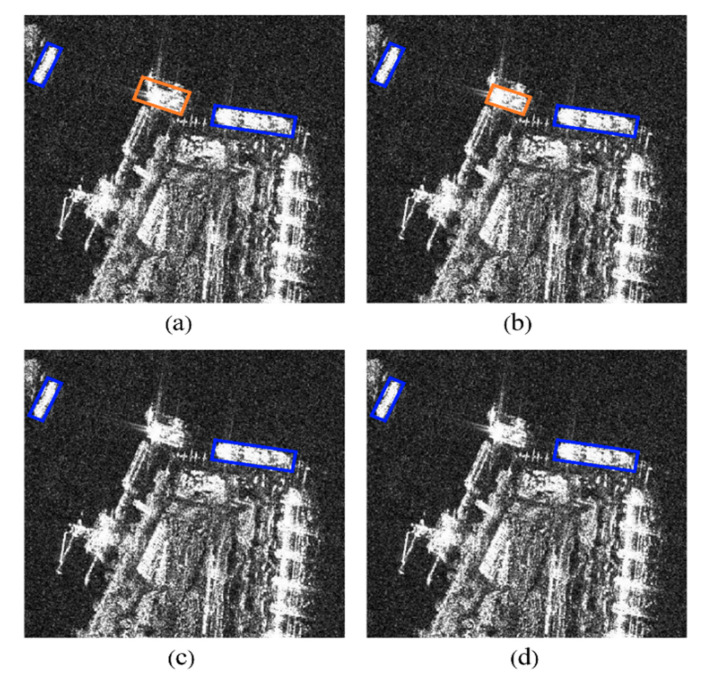
The effect of FEM. (**a**) The detection result of the experiment setting of NO CBAM and NO FEM. (**b**) The detection result of the experiment setting of YES CBAM and NO FEM. (**c**) The detection result of the experiment setting of NO CBAM and YES FEM. (**d**) The detection result of the experiment setting of YES CBAM and YES FEM. The blue rectangles indicate detected ships. The orange rectangles indicate the false alarms.

In order to verify the effectiveness of FEM, a contrast experiment is performed on the SSDD dataset. The detection results of the selected typical complex scenes are shown in Figure 20. It can be seen from Figure 20 that FEM shields islands and reefs that are very similar to ships, and reduces the false detection rate of ships. FEM improves AP by 1.09%. The detection results show that FEM enhances the foreground features and reduces the interference of complex backgrounds on ships.

## 6. Conclusions

The paper proposes a lightweight anchor-free detector for ship detection called R-Centernet+, which can balance precision and speed well. CBAM-DLA34 is used to extract ship features with attention. FEM is adopted to introduce foreground features in advance to obtain a feature map with enhanced foreground. The detection head is designed to conduct regression for the center point coordinates, size, offset and rotation angle of the ships, and realize the rotation detection of ship targets. We conduct the ship detection experiment on SSDD and AIR-SARShip datasets, the AP is 95.11% and 84.89%, respectively, and we achieve a high detection speed with a value of 33 FPS. Through comparison with Faster-RCNN and Centernet, the performance of the proposed model is better than these state-of-the-art detection models, which proves the robustness and practicability of the model.

Inshore ship datasets and offshore ship datasets are established based on SSDD and AIR-SARShip datasets, respectively, to evaluate the effectiveness of reducing background interference of the proposed model. For the inshore ships, the AP of the proposed model on SSDD-inshore and AIR-SARShip-inshore reach 93.72% and 68.45%, respectively. For the offshore ships, the AP of the proposed model on SSDD-offshore and AIR-SARShip-offshore reach 97.84% and 87.43%, respectively. The detection performance of ships of various sizes is satisfactory. The results show that the model can detect ships of multi-scale in inshore and offshore scenes.

In future work, we will continue to improve FEM. We will use the instance segmentation method to mark the foreground area of each ship target, respectively, to achieve a more refined foreground enhancement. On the basis of distinguishing ship targets and complex backgrounds, we will further distinguish different ship targets to better detect densely arranged ships.

## Figures and Tables

**Table 1:** The SSDD dataset.

Parameter	Value
Sensors	TerraSAR-X, RardarSat-2, Sentinel-1
Resolution (m)	1~15
Polarization	HH, VV, VH, HV
Scene	Inshore, Offshore
Sea condition	Good, bad
Images Size (pixel)	196~524 × 214~668
Images	1160
Ships	2578

**Table 2:** Statistical results of multiscale ships in SSDD.

Dataset	Ship Size		
	Small	Medium	Large
SSDD	1877	559	142

**Table 3:** SSDD-inshore and SSDD-offshore.

Datasets	Images	Ships
SSDD-inshore	227	668
SSDD-offshore	933	1910
SSDD	1160	2578

**Table 4:** AIR-SARShip dataset.

Parameter	Value
Sensors	GF-3
Resolution (m)	1~3
Polarization	Single
Scene	Inshore, Offshore
Sea condition	Good, bad
Images Size (pixel)	3000 × 3000
Images	31
Ships	1585

**Table 5:** Statistical results of multiscale ships in AIR-SARShip.

Dataset	Ship Size		
	Small	Medium	Large
AIR-SARShip	318	1094	173

**Table 6:** AIR-SARShip-inshore and AIR-SARShip-offshore.

Datasets	Images	Ships
AIR-SARShip-inshore	168	396
AIR-SARShip-offshore	551	1189
AIR-SARShip	719	1585

**Table 7:** Experimental results on different weight assignments.

$\lambda_{p}$	$\lambda_{s}$	$\;\lambda_{o}\;$	$\lambda_{a}$	$\lambda_{f}$	AP (%)
1.0	0.1	1.0	0.1	0.1	94.83
1.0	0.1	1.0	0.1	1.0	95.11
1.0	0.1	1.0	1.0	0.1	79.32
1.0	0.1	1.0	1.0	1	81.18

**Table 8:** Experimental results on the SSDD dataset.

Datasets	Models	Precision	Recall	$AP\;(AP_{50})$	$AP_{S}$	$AP_{M}$	$AP_{L}$
SSDD	R-Centernet+	95.21	95.58	95.11	93.01	97.87	98.52
	Centernet	93.44	94.28	93.82	92.15	96.08	96.14
	Faster-RCNN	89.48	89.80	88.96	86.95	89.53	87.02
SSDD-inshore	R-Centernet+	93.64	92.65	93.72	92.05	94.53	94.92
	Centernet	91.50	92.11	92.08	90.43	92.66	94.08
	Faster-RCNN	87.92	87.42	86.98	85.91	87.55	85.39
SSDD-offshore	R-Centernet+	96.75	97.85	97.84	97.24	99.83	99.72
	Centernet	94.62	94.85	95.32	93.07	96.96	97.26
	Faster-RCNN	89.42	91.18	90.05	88.84	91.36	89.78

**Table 9:** Experimental results on the AIR-SARShip dataset.

Datasets	Models	Precision	Recall	$AP\;(AP_{50})$	$AP_{S}$	$AP_{M}\;$	$AP_{L}$
AIR-SARShip	R-Centernet+	86.99	86.08	84.89	75.41	89.76	70.44
	Centernet	83.07	85.10	83.71	74.87	88.35	70.12
	Faster-RCNN	79.96	83.33	79.18	73.96	84.58	67.33
AIR-SARShip-inshore	R-Centernet+	72.92	76.09	68.45	67.77	83.18	63.91
	Centernet	71.97	75.70	66.83	66.04	82.97	62.88
	Faster-RCNN	80.39	55.16	58.72	56.38	69.86	62.02
AIR-SARShip-offshore	R-Centernet+	85.45	88.68	87.43	83.24	90.19	70.84
	Centernet	85.23	86.27	85.56	80.29	89.51	70.03
	Faster-RCNN	84.73	83.04	83.16	78.36	86.82	69.23

**Table 10:** Comparisons of detection performance with state-of-the-art models on SAR images.

Models	Backbone	SSDD	AIR-SARShip	FPS
Faster-RCNN	Resnet34	88.96	79.18	14
Centernet	DLA34	93.82	83.71	36
R-Centernet+	CBAM-DLA34	95.11	84.89	33

**Table 11:** Model comparisons with state-of-the-art models.

Models	Parameters	GFLOPs	Model Size (MB)
Faster-RCNN	192,764,867	23.93	539.06
Centernet	16,520,998	28.28	75.15
R-Centernet+	17,100,223	30.64	77.92

**Table 12:** Ablation experiments and results.

CBAM	FEM	Precision	Recall	AP (%)
No	No	92.05	92.63	93.82
Yes	No	94.52	93.62	94.62
No	Yes	94.37	94.58	94.91
Yes	Yes	95.21	95.58	95.11
